# A comparative assessment of mandible shape in a consomic strain panel of the house mouse (*Mus musculus*) - implications for epistasis and evolvability of quantitative traits

**DOI:** 10.1186/1471-2148-11-309

**Published:** 2011-10-19

**Authors:** Louis Boell, Sona Gregorova, Jiri Forejt, Diethard Tautz

**Affiliations:** 1 Max-Planck Institut für Evolutionsbiologie, August-Thienemannstrasse 2, 24306 Plön, Germany; 2 Institute of Molecular Genetics, Academy of Sciences of the Czech Republic, 14220 Prague, Czech Republic

## Abstract

**Background:**

Expectations of repeatedly finding associations between given genes and phenotypes have been borne out by studies of parallel evolution, especially for traits involving absence or presence of characters. However, it has rarely been asked whether the genetic basis of quantitative trait variation is conserved at the intra- or even at the interspecific level. This question is especially relevant for shape, where the high dimensionality of variation seems to require a highly complex genetic architecture involving many genes.

**Results:**

We analyse here the genetic effects of chromosome substitution strains carrying *M. m. musculus *chromosomes in a largely *M. m. domesticus *background on mandible shape and compare them to the results of previously published QTL mapping data between *M. m. domesticus *strains. We find that the distribution of genetic effects and effect sizes across the genome is consistent between the studies, while the specific shape changes associated with the chromosomes are different. We find also that the sum of the effects from the different *M. m. musculus *chromosomes is very different from the shape of the strain from which they were derived, as well as all known wild type shapes.

**Conclusions:**

Our results suggest that the relative chromosome-wide effect sizes are comparable between the long separated subspecies *M. m. domesticus *and *M. m. musculus*, hinting at a relative stability of genes involved in this complex trait. However, the absolute effect sizes and the effect directions may be allele-dependent, or are context dependent, i.e. epistatic interactions appear to play an important role in controlling shape.

## Background

The evolution and genetic architecture of shape is still poorly understood, but quantitative genetic studies based on morphometric approaches promise to lead a significant step forward [1]. The mouse mandible is a well-established model for studying the quantitative trait genetics of shape [2-11] as well as the analysis of general principles of wild type variation and evolution [12-14]. Given this quantitative genetics focus, the mouse mandible may also be a good model to address general questions of the nature and interactions of quantitative trait loci (QTL). This includes in particular the number and effect sizes of loci, the conservation of QTL across species and the prevalence of epistatic interactions.

In the classic infinitesimal model of quantitative genetic variation with mostly additive effects, one could expect relatively fast evolutionary divergence of the genetic architecture underlying phenotypic variation, since changes in one locus could easily be compensated by changes in another one. Although this is an issue of fundamental interest, only few studies have specifically addressed the question of evolutionary conservation so far [15].

There is some evidence pointing towards the existence of smaller sets of conserved "trait loci" underlying variation in some traits. For example, a conservation of major effect loci is generally implied in comparisons of medically relevant traits between mice and man. Furthermore, theoretical and empirical studies of metabolic flux control [16-19] suggest that variation at certain enzymes in metabolic pathways will produce most variation in flux, i.e. the propensity of some genes to produce phenotypic variation may be especially high. The same notion is supported by the empirical finding that QTL for univariate traits at certain genomic positions are recurrently identified across studies using different crosses of the same species [19-22], or sometimes even across species [23,24]. For example, a study on the comparison of atherosclerosis QTLs in humans and mice showed that 17 out of 27 loci mapped in humans correspond to homologous mouse QTLs [23], although there remains some uncertainty about the validity of this comparison [15]. But little is known so far about the conservation of QTLs for multivariate traits, such as shape. Because QTL studies of mandible shape have so far focused exclusively on mapping populations originating from a LG/J versus SM/J intercross, analysis of genetic variation between another set of mouse strains offers the opportunity to assess the conservation of the genetic basis of shape variation.

A topic closely related to this issue is the background-dependency of QTL effects, or epistasis. If the same loci or genomic regions recurrently exhibit large effect sizes on certain traits, this would mean that the relative effect sizes of QTLs on specific characters are usually background-independent. However, QTL-background interactions could still influence the specific phenotypic manifestation of genetic differences at QTLs, namely with regard to absolute effect sizes and the distribution of pleiotropic effects (multivariate direction of effects). This is likely to be relevant for complex, inherently multidimensional phenotypes such as shape, where the developmental processes affected by QTL variation are likely to affect multiple, albeit closely related "traits" (e.g., landmarks). Epistatic interactions for skull and/or mandible shape have been studied so far using both intercross QTL mapping designs [3] and a recombinant congenic strain panel [11]. Both studies found significant epistasis. However, this topic deserves further attention. The power to detect physiological epistasis using intercross designs is limited, because the complex patterns of marker and QTL segregation in an intercross mapping population reduce the power to detect epistasis between pairs of loci, since individual genotypes are not replicated and since multiple epistatic interactions in recombinant individuals may cancel each other out. Therefore, the amount of physiological epistasis is likely to be underestimated in such experiments. Congenic strains, which replicate individual genotypes and isolate small fractions of the genome on a uniform genetic background, are potentially a more powerful resource to assess the degree of epistasis. Similarly as congenic strains, chromsosome substitution strain (CSS) panels offer a powerful opportunity to detect physiological epistasis [25]. CSS panels consist of a number of strains derived from a receptor and a donor strain, where in each strain one chromosome of the receptor strain has been replaced by the corresponding chromosome of the donor strain.

According to the differences between a classical QTL experiment and a CSS panel, one could expect that it will be difficult to detect similarities in the genetic basis of quantitative traits in comparisons between such experiments, because the CSS effects are expected to represent unique genetic combinations including the possibility of strong epistatic interactions, whereas a QTL experiment measures additive effects of loci as average effects over many different combinations of genetic background.

We use here a morphometric analysis of mandible shapes in a CSS panel that was derived by introgressing single chromosomes from PWD, representing *M. m. musculus, *into the genomic background of C57BL/6J, representing mostly *M. m. domesticus *[26]. We were particularly interested to ask whether relative effect sizes and effect directions of genetic variation are comparable to those that were determined by QTL studies conducted with strains coming from one of the subspecies only (*M. m. domesticus*). Hence, we compare our results to the QTL mapping study by Leamy et al. [5] using an almost identical set of landmarks.

We find that there is indeed a correlation between effect sizes between the QTL and the CSS results, although not for all loci. Effect directions were, however, not correlated, suggesting major allelic differences. When we combine the effects of all CSS strains, this leads to an extreme hypothetical phenotype outside the range of known natural variation. This confirms the notion of major epistasis effects uncovered through the analysis of consomic strains [25]. Our results thus provide insights into the genomic architecture and evolution of multivariate quantitative traits.

## Results

### Chromosomal effects on mandible shape in the consomics panel

We analyzed the shape differences between mandibles of the C57BL/6J strain and the PWD/Ph strain, as well as between the chromosome substitution strains (CSS) derived from these strains using C57BL/6J as a receptor strain and PWD as a donor strain [26].

In separate pairwise discriminant function analyses, 25 of 28 PWD chromosomes or chromosome fragments had a significant effect on mandible shape at the 5% level based on a permutation test of 10,000 runs for the T-square statistic after adjusting for multiple testing using sequential Bonferroni correction. Leave-one-out-cross validation tests supported the notion of a high confidence for the specific effect in each strain, with on average less than a quarter of specimens that could not be unambiguously assigned to their strain (Table 1).

**Table 1:** Statistical comparisons of strains.

Strain	N (F/M)	Procrustes ANOVA			DFA sex			DFA strain		
		**Strain**	**Sex**	**Strain × Sex**	**p- value**	**% misass.**	**Proc. dist.**	**p- value**	**% misass.**	**Proc. dist.**

C57BL/6J	18 (9/9)				0.8788	22	0.0227			

PWD	20 (6/14)	<0.0001*	0.7181	<0.0001*	0.9790	45	0.0152	<0.0001*	0	0.0442

Chr1^a^	18 (8/10)	0.0006*	0.0242	0.0593	0.7270	11	0.0213	<0.0001*	22	0.0245

Chr2^a^	17 (9/8)	<0.0001*	0.0151	0.0771	0.9820	65	0.0217	<0.0001*	14	0.0279

Chr3^a^	17 (9/8)	0.0004*	0.6075	0.0205	0.9350	47	0.0121	<0.0001*	17	0.0271

Chr4^a^	17 (7/10)	0.0002*	0.0098	0.2047	0.9615	53	0.0147	<0.0001*	0	0.0222

Chr5^a^	17 (8/9)	0.0443	0.3358	0.0003*	0.7624	12	0.0229	0.0010*	29	0.0219

Chr6^a^	18 (7/11)	0.0078	0.7694	0.0014*	0.9990	72	0.0138	<0.0001*	22	0.0248

Chr7^a^	17 (6/11)	0.003*	0.8293	<0.0001*	0.9830	53	0.0252	<0.0001*	26	0.0344

Chr8^a^	14 (7/7)	0.0002*	0.0025*	0.223	0.8990	14	0.0287	<0.0001*	42	0.0263

Chr9^a^	17 (10/7)	0.0496	0.017	0.0085	0.8576	6	0.0249	0.0010*	14	0.0182

Chr10-d^a^	18 (10/8)	<0.0001*	0.912	0.0003*	0.9960	50	0.0114	<0.0001*	14	0.0367

Chr10-m^a^	15 (8/7)	0.4782	0.08	0.0128	0.7760	27	0.0203	0.085	39	0.0132

Chr10-p^a^	17 (2/15)	0.0355	0.01	0.0058	0.8202	29	0.0294	<0.0001*	34	0.0197

Chr11-d^a^	19 (8/11)	0.003*	0.8586	0.0002*	0.9720	58	0.0114	<0.0001*	3	0.0257

Chr11-m^a^	17 (9/8)	0.0054	0.0019*	0.4491	0.9942	42	0.0205	0.0020*	50	0.0174

Chr11-p^a^	16 (11/5)	0.0481	0.6601	0.006	0.9991	50	0.0141	<0.0001*	20	0.02

Chr12^a^	19 (9/10)	0.0005*	0.2786	0.052	0.9862	53	0.012	<0.0001*	19	0.024

Chr13^a^	18 (6/12)	0.0657	0.8668	0.0004*	0.9513	39	0.0121	<0.0001*	31	0.0195

Chr14^a^	19 (10/9)	0.4924	0.1372	0.0027*	0.8681	32	0.0196	0.03	35	0.0132

Chr15^a^	18 (8/10)	0.0006*	0.029	0.1826	0.9193	39	0.0158	<0.0001*	22	0.0223

Chr16^a^	16 (8/8)	0.0005*	0.0173	0.1344	0.9868	44	0.0158	<0.0001*	17	0.0217

Chr17^a^	16 (8/8)	0.0048	0.0464	0.0858	0.9905	50	0.016	<0.0001*	17	0.0198

Chr18^a^	17 (8/9)	0.0004*	0.0043*	0.4045	0.9940	59	0.0181	<0.0001*	17	0.0212

Chr19^a^	17 (10/7)	0.0235	0.0055*	0.303	0.9983	41	0.0163	0.0090*	26	0.0155

X-d^a^	17 (10/7)	0.7137	0.9467	<0.0001*	0.9262	53	0.0184	0.029	34	0.0143

X-m^a^	19 (10/9)	0.4367	0.4556	<0.0001*	0.8389	41	0.0155	0.0050*	19	0.0149

X-p^a^	18 (9/9)	0.0005*	0.2022	0.1635	0.9974	50	0.0108	<0.0001*	28	0.0231

Y^a^	16 (9/7)	0.0012*	0.2378	0.0011*	0.9401	25	0.0206	<0.0001*	26	0.0262

Mit^a^	16 (8/8)	0.0542	0.105	0.17	0.9918	50	0.0121	0.0090*	59	0.0158

Numbers of specimens per strain (Females/Males), P-values for effects of strain sex and strain × sex interaction after pairwise two-way Procrustes Anova comparisons of the consomic strains against C57BL/6J, P-values for differences in Procrustes mean shape after pairwise DFAs of the consomic strains by sex and against C57BL/6J, per cent of specimens incorrectly assigned in cross-validation tests of the DFAs, and Procrustes distances between sexes and between C57BL/6J and the consomic strains. Stars indicate significant p-values after sequential Bonferroni correction assuming a general significance level of 0.05.

^a ^The full nomenclature of the strains is C57BL/6J-Chr #^PWD/Ph^/ForeJ, with # representing the chromosome number or designation respectively

Analyses of sexual dimorphism and sex effects yielded inconclusive results. Using discriminant function analyses between sexes within each strain (Table 1), we found no significant sexual dimorphisms. In contrast, two-way Procrustes ANOVA for strain and sex on pairwise comparisons of each CSS strain against C57BL/6J yielded significant sex or sex × strain interaction effects for some strains (Table 1). These results are more difficult to interpret than the results on differences between strains, probably due to low sample sizes for each sex, high variation between individuals and a high number of degrees of freedom (see discussion). In the following we do not differentiate between sexes, making our comparative analysis more conservative.

**Figure 1:**
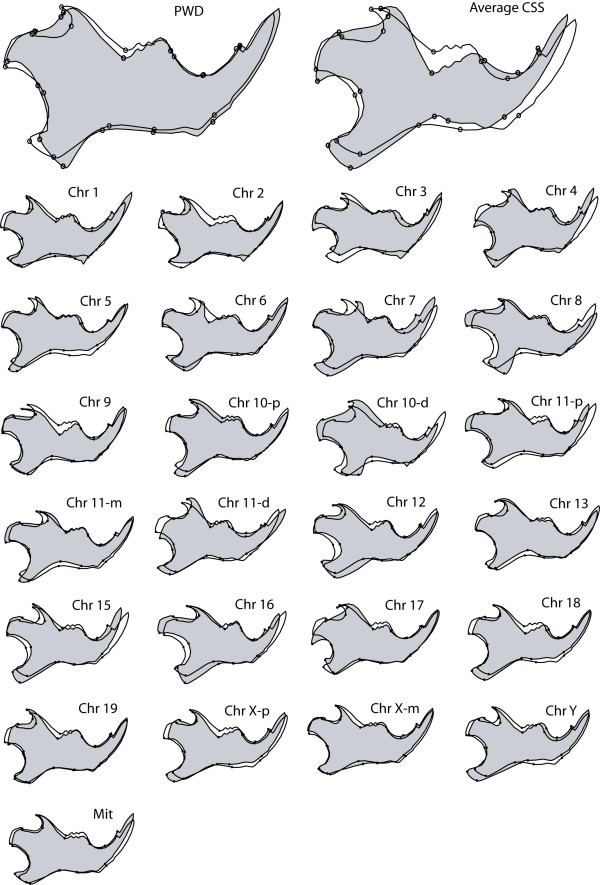
**Procrustes mean shape differences.** Differences are depicted with respect to C57BL/6J (black outlines) and PWD (exaggerated × 2), the average consomic shape (exaggerated × 20) and the CSS shapes for each strain (exaggerated × 5) (grey areas respectively).

Figure 1 provides a visualization of the shape changes of PWD, the average consomic shape and the individual consomic strains relative to C57BL/6J. The shape changes affect all parts of the mandible, and no simple pattern emerges from visual inspection.

### Effect sizes

We used the results of the QTL study by Leamy et al. [5] for comparison. This study was based on an analysis of a F3 population derived from two inbred strains of *M. m. domesticus*. Effect sizes in this study were given as vector lengths of shape changes, which correspond to Euclidean/Procrustes distances between genotypes in the tangent coordinate space. The chromosome-wide additive effect sums (vector lengths of within-chromosome QTL effect vector sums) are therefore directly comparable to the Procrustes distances between C57BL/6J and the CS strains.

**Figure 2:**
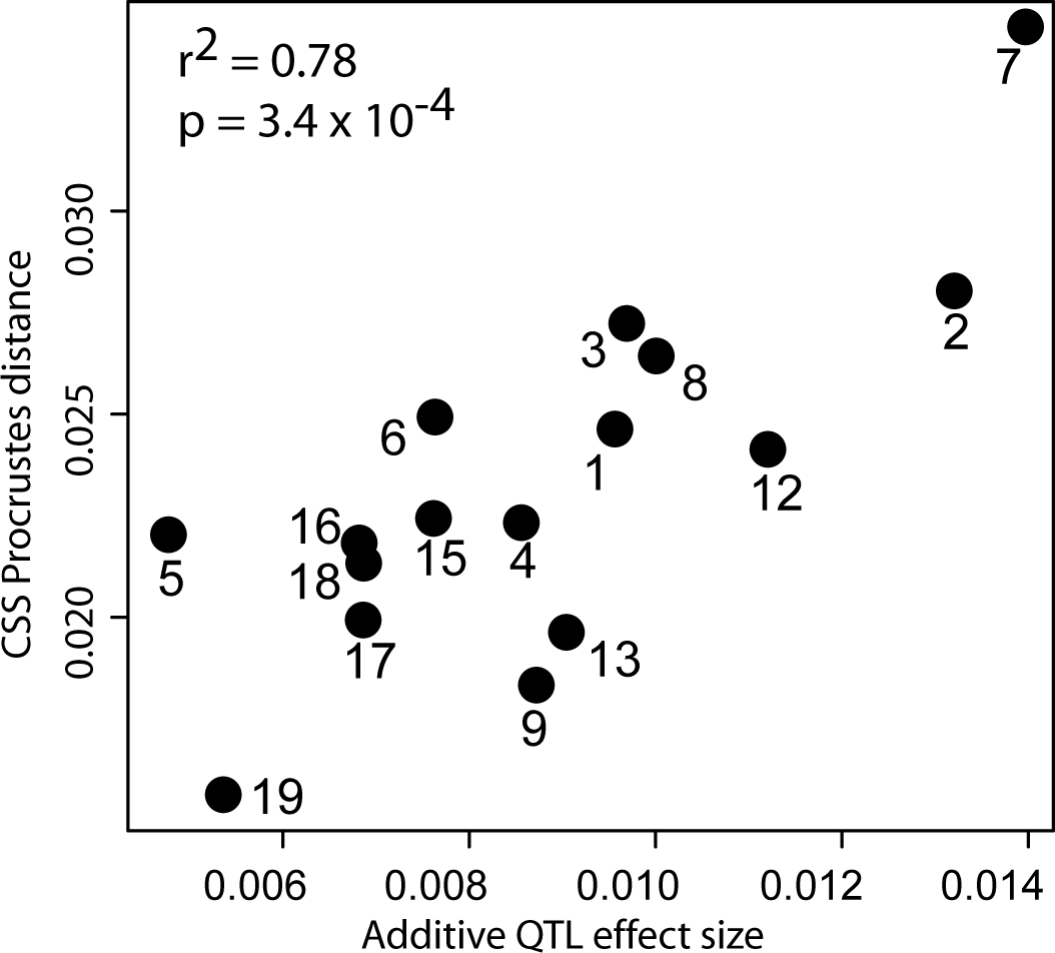
**Correlation between Procrustes distances in the consomic panel (CSS) and additive QTL effects from **[5]. The QTL effects are shown as Procrustes distances between chromosome-wide homozygotes. Numbers in the plots designate Chromosomes.

For chromosomes 1-9, 12, 13 and 15-19, a comparison of chromosomal effect sizes between the CSS panel and the QTL results from [5] is shown in Figure 2. QTL and CSS effect sizes are highly correlated (r = 0.78, p = 0.00034). The absolute effect sizes for the QTL are on average 37% of the CSS effect sizes.

Chromosomes 10 and 11, which could not be introgressed into the C57BL/6J background as a whole chromosome, but only as fragments included in recombinant chromosomes [26], were found to behave differently and were thus analyzed separately. A comparison of QTL and CSS effects for subregions of these chromosomes is shown in Table 2. For these chromosome fragments, there appears to be no positive correlation between QTL and CSS effect sizes (r = -0.42, p = 0.48), but this is likely to be due to the small number of data points.

**Table 2:** QTL and CS effects for subregions of chromosomes 10 and 11 in units of Procrustes distance (between homozygotes).

QTL	QTL effect	CS effect	CS
Sh10.1	0.0064	0.0132	10 m
Sh10.2	0.0060	0.0367	10 d
Sh11.1	0.0112	0.0200	11 p
Sh11.1	0.0112	0.0174	11 m
Sh11.2	0.0078	0.0257	11 d

Under the infinitesimal model, congruence between QTL and CSS effect sizes could simply be related to the length of the chromosomes, with longer chromosomes harbouring more quantitative trait genes. Only two QTL are detected per chromosome in [5], but this could be due to limits of power and resolution. Indeed, both QTL and CSS effect sizes are correlated to chromosome length in Mb (QTL effects: r = 0.55, p = 0.028; CSS effects: r = 0.64, p = 0.007). In order to test whether chromosome length is the underlying variable causing the congruence between QTL and CSS effects, we regressed both sets of effects on chromosome length and measured the correlation between the residuals. The correlation between the residuals was still 0.67 (p = 0.004); therefore, length is not the variable that explains this.

### Effect directions

**Figure 3:**
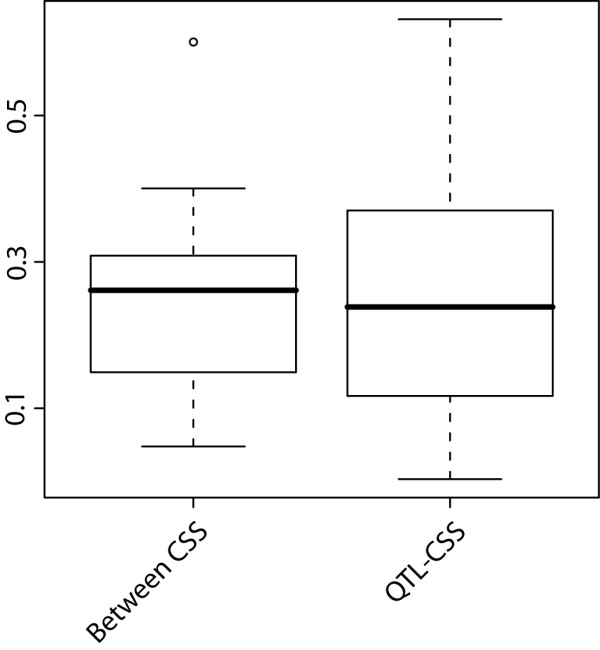
**Magnitudes of vector correlations among C57BL/6J-versus-CS strain Procrustes mean shape difference vectors and between them and the corresponding chromosome-wide additive QTL effects in **[5].

Since relative effect sizes appear to be conserved between the CSS panel and the QTL study, one might expect that the directions of shape change associated with genetic differences in these chromosomes could also be conserved. We assessed this using vector correlation (Pearson correlation between vectors) between the CSS and QTL shape change vectors. Because the elements of shape change vectors derived from biological data are unlikely to be comparable to random vectors of the same length, we could not use conventional significance testing procedures for assessing biological significance of these correlations. Therefore, we used among-CSS-effect vector correlations as a null distribution to assess the overall significance of chromosome-wise correlations between QTL and CSS effects. Vector correlations between chromosome-wide additive QTL and CSS effects are compared to among-CSS-effect vector correlations in Figure 3. There is no significant difference between the means of both sets of correlation magnitudes, indicating that QTL and CSS effect directions show no special similarity at the chromosomal level.

### Context-dependency of chromosomal effects

**Figure 4:**
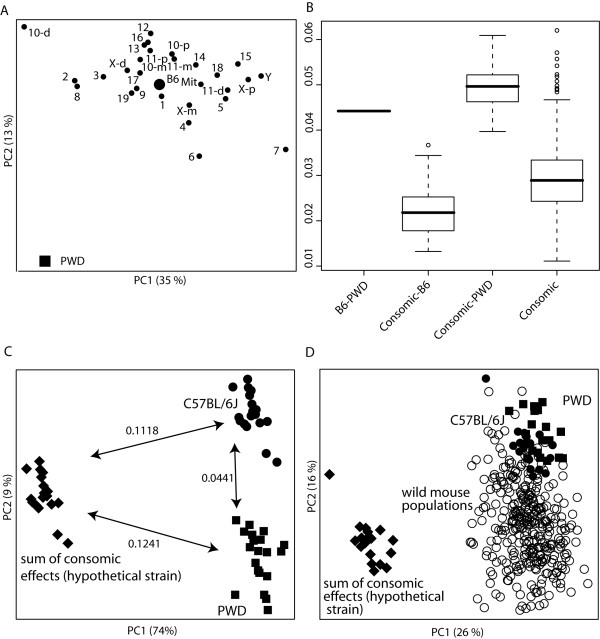
**Distance assessments between consomic strains and founder strains.** A) Scatterplot of the first two PCs of a principal components analysis including strain mean shapes of PWD (large square), C57BL/6J (large dot) and the consomic strains (small dots). B) Pairwise Procrustes distances between PWD, C57BL/6J and the CS. C) Scatterplot of the first two PCs of a principal components analysis including PWD (squares), C57BL/6J (dots) and an artificial "strain" (diamonds) created by adding the sum over all C57BL/6J-CS strain mean Procrustes coordinates-differences to the C57BL/6J individual shapes. This "B6 + sum of consomic effects" population represents the hypothetical phenotypes which would occur if the individual effects of all chromosomes acted together in a purely additive fashion. Numbers on arrows indicate Procrustes distances between the strains. D) as before, but including the wild mouse populations from [14] (circles), indicating the range of natural variation of house mouse mandible shape (see text).

If the chromosomal effects were context-independent in absolute size and direction, then the shape differences between C57BL/6J and the consomic strains should add up to the difference between C57BL/6J and PWD. We tested three assumptions according to this expectation:

First, the position of the consomic strains in the shape space should cluster around the C57BL/6J-PWD-axis. A scatterplot of the first two PCS of a PCA on the Procrustes coordinates of the strain mean shapes of C57BL/6J, PWD and the CS strains shows that this is not the case (Figure 4A). Instead, the scatter of the consomic strains around C57BL/6J appears to be independent of the C57BL/6J-PWD-axis. Second, the Procrustes distances between PWD and the CS strains should be smaller than the distance between C57BL/6J and PWD. In fact, they are mostly even larger (Figure 4B). Third, an artificial "strain" created by adding the sum of all 28 CS strain-C57BL/6J shape differences to C57BL/6J individual shapes should come to lie at a similar distance and in a similar direction from C57BL/6J as PWD. Figure 4C shows that this artificial "strain" lies at a far distance from both C57BL76J and PWD, and in a different direction from C57BL/6J than PWD. The absolute effect sizes and the direction of the chromosomal effects are therefore not context-independent. In fact, a PCA comparison of the mandible shapes of C57BL/6J, PWD and the hypothetical strain (Figure 4D) to a survey of wild populations that represents the natural range of variation in mandible shape [14] shows that the shape of the latter constitutes a "monster shape" far outside the range of natural variation.

## Discussion

Our results provide insights into evolutionary patterns at multivariate quantitative trait loci, as well as the general genetic architecture of mandible shape. Little was known so far whether this is conserved between distinct populations and species, and to which degree magnitude and direction of genetic effects on shape are dependent on genetic background.

### Sexual dimorphism

Our results on sexual dimorphism are somewhat contradictory, with discriminant function analysis finding no significant dimorphisms, while ANOVA finds significant sex or strain × sex interaction effects for 15 strains. This is likely due to the combination of low sample sizes for each sex with a high number of degrees of freedom (24 df). This may on the one hand reduce the power for DFA to find dimorphisms, and on the other hand cause false positives due to sampling error in the ANOVA. It is surprising to find apparently significant effects for so many strains, because sexual dimorphism of mandible shape in mice has previously not been frequently detected [14], and only one sex-specific QTL was found in [5] to which we compare our results. This aspect should therefore be revisited using higher sample sizes. Sex effects, if present, may interfere with our comparison between the CSS panel and the QTL study in [5]. If we find similarity in spite of this, it means that the respective pattern is relatively robust.

### Comparison of relative effect sizes

Both the QTL study [5] and our investigation of the C57BL/6J-PWD CSS panel map significant effects on shape to most chromosomes. We used correlations across chromosomes between the QTL effect sizes (Procrustes distances between chromosome-wide homozygotes), and CSS effects (Procrustes distances between CS strains and C57BL/6J), to compare the distributions of relative effect sizes. Except for chromosomes 10 and 11, which are fragmented in the CSS panel, we find significant similarity of the distribution of effect sizes across chromosomes in both studies. This suggests good conservation of general aspects of the genetic basis of shape variation.

This finding is especially surprising, since stronger epistatic effects in the CSS panel could have been expected to obscure such correspondences. It appears thus from our results that at least the chromosome-wide relative effect sizes are relatively independent of genetic background. One appealing explanation is that a set of major effect genes is responsible for effect sizes. It is now generally assumed that QTL allelic effects do not represent an infinitesimal effect distribution, but follow an exponential distribution [27] with a few loci with moderate to large effects, and increasingly larger numbers of loci with increasingly smaller effects [15]. There are possible biological explanations for this pattern at the population genetic [28] and the physiological [18] level. Since traditional QTL studies tend to uncover only the large effect loci, our study would address the question whether the loci making up the trunk of the distribution can be expected to remain large effect loci over evolutionary time. Alternatively, one could have imagined that loci with minor effects could have replaced major effect loci in different lineages, which would lead to an evolutionary turnover of genes involved in quantitative traits. Our results are less compatible with this hypothesis. One should, however, bear in mind that the genetic resolution of QTL studies is low, and the genetic resolution in a CSS panel is even lower. Therefore, even though we find congruence between both analyses, further research will be needed to decide whether this is indeed based on identity of the underlying genes or on other as yet unknown factors or mechanisms. For example, it is known that single large-effect QTL resulting from low-resolution experiments can break down into multiple QTL of smaller effect in subsequent high-resolution QTL experiments [15]. Also, the fact that we could not find correspondence of relative effect sizes for the subconsomic strains containing the fragments of chromosomes 10 and 11 to QTL effects suggests additional factors that need further study.

### Comparison of effect directions

We used vector correlations between additive, dominant and sum QTL effects on the one hand and CSS shape change vectors on the other hand to assess similarity of effect directions between the QTL and the CSS results on a chromosome-by-chromosome basis. Compared to the similarity of chromosomal effects within the CSS panel, which we used as a null distribution, we found no significant similarity of effect directions between the studies. This result suggests that in contrast to effect size, effect directions are not comparable between the groups. However, this is expected since we are evidently not comparing the same alleles. There are on average 5 replacements per kb between the subspecies [29], i.e. it is likely that new alleles have evolved since their split. Hence, although the genetic loci relevant for shape, and thus the underlying genetic architecture, may be conserved, different alleles at these loci can still be involved in evolutionary divergence.

### Non-additivity

Under a functionally additive model, the effects of substituting individual chromosomes should add up across the CSS panel to yield the difference between donor and receptor strain. However, a study on 90 medically relevant univariate traits in mouse and rat CSS panels found little additivity [25]. These authors suggest pervasive epistasis causing these effects. Therefore, we expected to find high amounts of epistasis in our CSS panel. In agreement with this, the sum of the chromosomal effects across the CSS panel is several times larger than the difference between donor and receptor strain. Epistasis with respect to shape would mean that the different inputs from developmental pathways do not simply add up in their effects, but can lead to rather different outcomes in dependence of the genetic context. A comparison of the hypothetical shape generated by adding all chromosomal effects across the PWD to the C57BL/6J shape with the range of natural variation (Figure 4D) shows that the genetic variation unravelled in the CSS panel would engender a highly abnormal phenotype if the effects acted cumulatively in an additive fashion.

Genetic background also affects the direction of chromosomal effects. Thus, the net chromosomal effect across the CSS panel lies in a different direction than the C57BL/6J-PWD difference (Figures 1, 4C). This indicates that QTL mapping to the same genomic region can have different phenotypic effects dependent on the genetic background. Such epistasis between shape QTLs could greatly enhance the number of degrees of freedom of genetic shape variation, in contrast to a scenario where at least one gene would have to be specific for variation in a given direction.

## Methods

### Samples

We analysed 518 mice from the C57BL/6J-Chr #^PWD/Ph^/ForeJ chromosome substitution (consomic) strain panel [26] (Table 1). All mice were reared in the Institute of Molecular Genetics, Academy of Sciences of the Czech Republic, Prague (affiliation of JF and SG). Mice were sacrificed between 75 and 95 days of age (85 days on average). Mouse heads were preserved in Ethanol.

### Data acquisition

Specimens were scanned with a tomograph (microCT - VivaCT 40, Scanco, Bruettisellen, Switzerland). Two-dimensional x-ray images of right hemi-mandibles were obtained from micro-CT data as described in [14]. Landmarks were digitized once for each specimen on the images using tpsDig2 [34] and tpsUtil [35], producing a set of 28 raw coordinates for each specimen (the estimation error for Procrustes distances due to measurement using our method is known from previous analyses to be 4% on average [14]).

### Morphometric and statistical analyses

Geometric morphometric analyses were performed in MorphoJ 1.02b [36], following general principles of geometric morphometrics see [37].

We used discriminant function analysis (DFA) of the Procrustes coordinates to quantify distinctness between each consomic strain and the receptor strain C57BL/6J. For these analyses, a separate Procrustes superimposition was performed for each strain together with C57BL/6J. To evaluate the significance of shape differences between CSS and C57BL/6J, we used the permutation test of 10,000 runs for the T-square statistic and the leave-one-out-cross-validation implemented in MorphoJ (for details see documentation of MorphoJ). P-value thresholds for significance assessment were adjusted *post hoc *using the sequential Bonferroni-Holm procedure.

Differences between sexes within each strain were assessed using DFA and two-way ANOVA. For the DFA, a separate Procrustes superimposition was performed for each strain and DFA by sex was performed within each strain, such that confounding strain effects were avoided. Two-way ANOVA with strain and sex as fixed effects was carried out for each CSS strain on a dataset consisting of the respective strain and C57BL/6J.

Finally, to quantify the CSS effects in terms of Procrustes distances between C57BL/6J and each CSS, we used CVA in MorphoJ on a dataset consisting of the entire CSS panel after a common Procrustes superimposition.

In order to compare our results with the QTL effects from [5], we needed to be able summarize the latter at a chromosome-wide level. The QTL effects in [5] are shape change vectors, which resulted from regression analyses of Procrustes coordinates on QTL genotype.

Therefore, we obtained these vectors from the authors and summarized chromosome-wide QTL effects as the sum vector of the additive QTL effect vectors within each chromosome. This was calculated by adding, for each chromosome, the corresponding 30 elements (x and y coordinate values for 15 landmarks) of the effect vectors of the two QTL on that chromosome, squaring and adding up the elements of the resulting vector, and multiplying the square root of that sum by 2 since the additive values represent 1/2 of the difference between homozygotes.

### Comparison of chromosomal effects

To compare the QTL effects in Leamy et al. [5] and our CSS results, we represent the QTL effects at a chromosome-wide level as the sum of the additive effects of all QTL per chromosome, which is the length of the vector sum of the additive effect Procrustes shape change vectors.

For chromosomes 1-9, 12, 13 and 15-19 we calculated the Pearson correlation across chromosomes between the additive QTL effect sizes per chromosome and the Procrustes distances between the CSS strains and C57BL/6J. We excluded chromosome 14, because it had no significant effect in the CSS panel. Furthermore, we chose to ignore the sex-specific QTL on chromosome 6, because we found no sexual dimorphism in the CSS for this chromosome. Chromosomes 10 and 11 were investigated separately, because they were introgressed as three separate fragments each in the CSS panel, and the resulting six strains are thus carrying recombinant chromosomes, which represents a special condition. For chromosomes 10 and 11, we assessed according to map locations of the CI intervals in [5] which of the strains carrying the introgressed fragments are expected to carry the QTL. We then compared the Procrustes distances between C57BL/6J and these fragment substitution strains to the corresponding QTL effect sizes in [5].

To test the influence of chromosome length as an underlying variable, we used linear regression of effect sizes on chromosome length. This was done in R [38].

To compare the directions of shape changes, we correlated each chromosomal additive QTL effect vector (the sum vector of the additive effect vectors of both QTL on each chromosome) with the Procrustes mean shape difference vector between C57BL/6J and the corresponding CS strain. For this purpose, we homologized landmarks between studies as follows: landmarks 1-14 in [5] correspond to landmarks 1-14 in our study. Pearson correlations were calculated in PAST [39]. To evaluate the significance of the resulting vector correlations, we also calculated pairwise correlations between all CSS effect vectors. This was used as a null distribution, because we cannot expect biological shape change vectors to be comparable to random vectors. The means of both distributions of correlation magnitudes (QTL-CSS and among-CSS) were then compared using a t-test.

To visualize pairwise shape differences between C57BL/6J and individual CS strain shapes, we averaged datasets containing C57BL/6J and the respective strain in MorphoJ by strain followed by PCA (principal components analysis), such that the shape change vector associated with the unique principal component represented the shape difference between the two shapes.

To assess whether chromosomal effects add up in magnitude and direction to the difference between donor (PWD) and receptor (C57BL/6J) strain, we calculated, for each CS strain, the difference between its Procrustes mean shape and the Procrustes mean shape of C57BL/6J. We then summed all difference vectors and added this difference sum vector to the Procrustes coordinates of each C57BL/6J individual, thus creating a hypothetical strain of C57BL/6J mice changed by the sum of all individual PWD chromosomal effects in the C57BL/6J background. This hypothetical strain was compared to C57BL/6J and PWD using CVA. Furthermore, we used PCA to compare PWD, C57BL/6J and the hypothetical strain to a survey of natural variation of mouse mandible shape [15].

## Conclusions

Our results suggest that parallel evolution based on the same QTLs, such as has been found for simpler traits in a variety of organisms [30], may also occur for shape. At the same time our results show that variation at the same QTLs may be associated with phenotypic variation in multiple directions, depending on allelic differences and epistatic interactions with other loci. Therefore, a limited number of important loci may be sufficient to produce a complex, multidimensional pattern of variation that could evolve in many directions. The fact that the individual effect sizes of the CS strains cannot simply be added up to obtain the shape of the donor strain suggests that there is a physiological limit to the phenotypic variation caused by modulation of developmental pathways through genetic variation at QTLs in a cumulative fashion. This type of "epistatic buffering" [25] might allow natural populations to accumulate genetic variation for complex traits, which could be converted into additive genetic variance and become available for selection in combination with founder events or strong population structure [31-33].

## Authors' contributions

LB and DT designed the experiment, LB did the measurements and the statistics, SG and JF raised the mice, LB and DT wrote the manuscript with input from SG and JF. All authors have read and approved the final manuscript.
